# Insight Into Systemic Lupus Erythematosus: Unveiling Central Retinal Artery Occlusion as an Initial Indicator

**DOI:** 10.7759/cureus.67276

**Published:** 2024-08-20

**Authors:** Malik Hasnat ul Hassan Khan, Haider Sarfaraz, Nida Khan, Muhammad Hamza Mushtaq, Syed Aftab Ahmad, Muhammad Abbas, Ihtisham Habib, Muhammad Amin Noor

**Affiliations:** 1 Internal Medicine, Medical Teaching Institute, Lady Reading Hospital Peshawar, Peshawar, PAK; 2 Internal Medicine, Medical Teaching Institute, Khyber Teaching Hospital Peshawar, Peshawar, PAK

**Keywords:** connective tissue disorder, systemic lupus erythematosis, micro-vasculopathy, central retinal artery occlusion (crao), sudden vision loss

## Abstract

Systemic lupus erythematosus (SLE) is a chronic autoimmune disorder with diverse clinical manifestations. Among these, ocular complications are notably prevalent, affecting up to one-third of patients. One rare but serious ocular complication of SLE is central retinal artery occlusion (CRAO), which can result in significant vision loss. We report a case of a young woman with sudden, painless vision loss in her right eye over two days. Fundoscopy confirmed CRAO, with no light perception in the affected eye and normal vision in the left eye. Physical examination revealed symptoms suggestive of a connective tissue disorder, including malar rash and Raynaud’s phenomenon. Laboratory tests confirmed SLE. Despite treatment with methylprednisolone, hydroxychloroquine, aspirin, and nifedipine, the patient’s vision did not improve. CRAO in SLE indicates severe retinal vasculopathy and has a poor prognosis. This case highlights the importance of considering SLE in patients with sudden vision loss and systemic symptoms, emphasizing early diagnosis and comprehensive management to prevent severe complications.

## Introduction

Systemic lupus erythematosus (SLE) is a multisystem chronic autoimmune disease with a relapsing and remitting course. The disease has varied presentations, ranging from mild mucocutaneous manifestation to multiorgan disease involvement [1]. The exact cause is unknown, but genetic, immunological, endocrine, and environmental factors are believed to trigger the body’s immune response, resulting in excessive pathogenic autoantibodies producing tissue and organ damage [2]. Women are ten times more at risk of developing SLE than men, with a female and male ratio of 9 to 1 [1]. SLE diagnosis is based on laboratory and clinical findings [3]. The treatment depends on the disease's severity and the organs involved [4]. Ocular complications are found in up to one-third of patients with SLE and can range from mild eye irritation to vision-threatening retinopathy [5,6]. Lupus retinopathy is the most vision-threatening ocular manifestation ranging from 3 to 29% of patients affected due to vasculitis of retinal arterioles and capillaries. The occlusion of vessels can result in retinal hemorrhage, exudate, and cotton wool spots. Occlusion of large arteries can result in life-threatening vision loss. Such cases are less prevalent with available treatment modalities [7].

## Case presentation

A young married female presented to our hospital outpatient clinic with chief complaints of loss of vision in her right eye. Vision loss was sudden in onset, non-progressive, and non-painful. The episode of vision loss occurred two days prior. Any head or eye pain did not accompany the vision loss. She had no significant past ophthalmologic history and no family history of coagulopathy.

**Figure 1:**
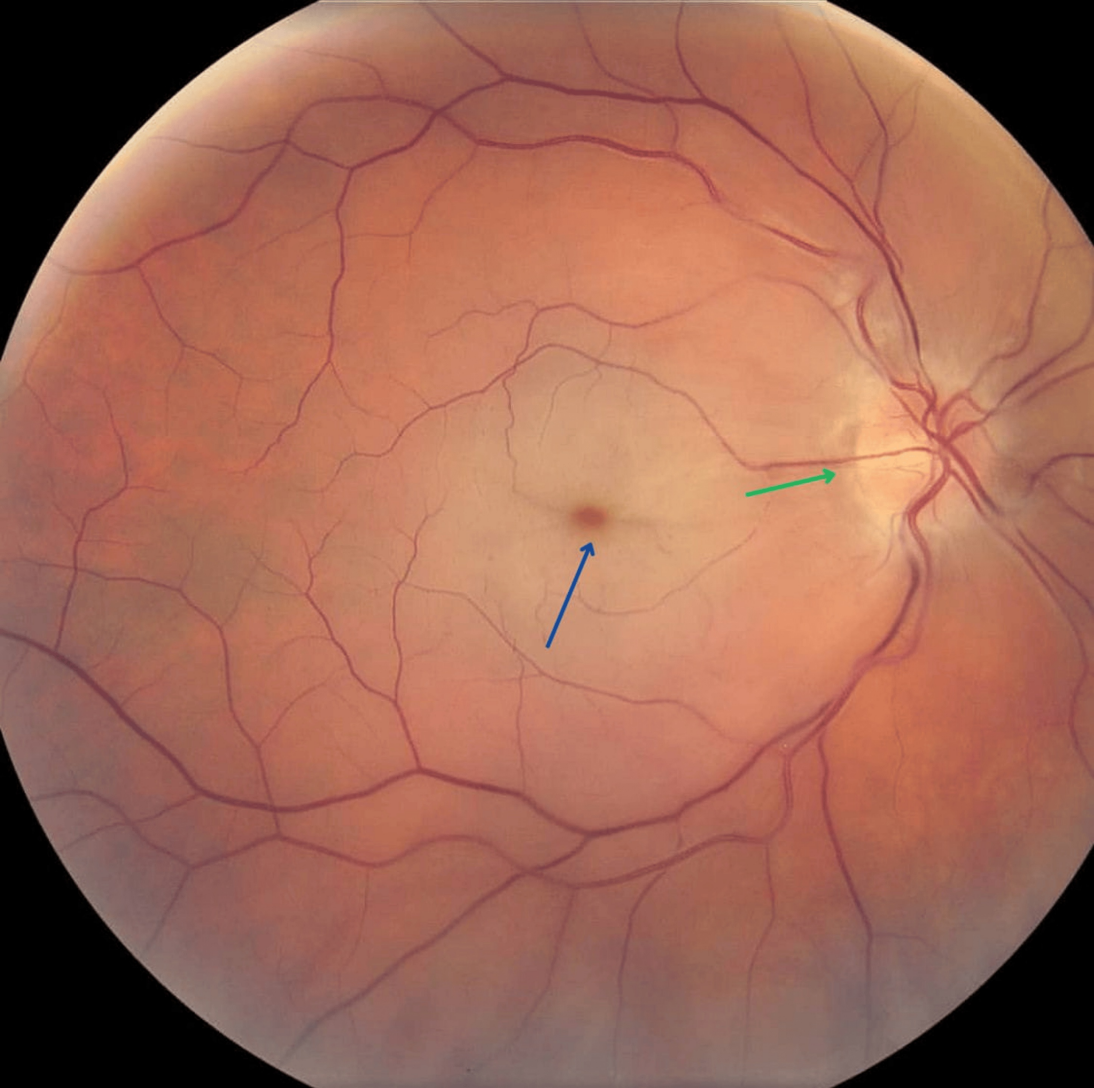
Fundus photograph showing cherry red macula (black arrow) and pale optic disc (green arrow).

The ophthalmologic examination showed that the right pupil was mid-dilated and non-reactive to light, but responded to an indirect light reflex. There was no ocular redness, discharge, or periorbital swelling. On visual acuity testing, her vision showed no perception of light (NPL) in the right eye and 20/20 in the left eye. Fundoscopy showed central retinal artery occlusion (CRAO) with a pale optic disk and cherry red spot (Figure 1).

A detailed physical examination of the patient revealed malar rash, alopecia, polyarthralgia, and Raynaud phenomena. The mentioned physical examination finding raised suspicion of underlying connective tissue disorder, and the patient was admitted to the hospital floor for further workup.

Blood tests were mostly normal, except for a hemoglobin level of 9.89 g/dL and an elevated creatinine level of 1.4 mg/dL (Table 1). Brain MRI, carotid Doppler, and echocardiogram were unremarkable. However, Doppler ultrasonography of the peripheral limbs revealed atherosclerotic changes. Urinalysis showed 1+ protein and 8-10 pus cells and 24-hour urinary protein was 423 mg/24 h (Tables 2, 3). The antinuclear antibody (ANA) screen was positive, and further testing revealed elevated anti-dsDNA antibodies, confirming the diagnosis of SLE. Consultation with the ophthalmology and rheumatology teams led to the initiation of treatment with intravenous methylprednisolone 500 mg daily for three days, along with oral hydroxychloroquine (HCQ), aspirin, and nifedipine. The antiphospholipid antibody screen was negative, and the patient's history did not reveal any miscarriages. Despite maximal pharmacotherapy, the patient showed little to no improvement in right-eye vision. She was discharged on tablet HCQ, tablet aspirin, and sunblock for her face. After a three-month follow-up, the patient reported marked improvement in fatigue and joint pain, but her right eye vision was still NPL.

**Table 1:** Basic lab investigations. ALT: alanine transaminase; ESR: erythrocyte sedimentation rate; HCV: hepatitis C virus; HBsAg: hepatitis B surface antigen.

Parameter	Value	Reference range
Total leukocyte count	7930/ul	4000-11000/ul
Hemoglobin	9.89 g/dl	11.5-17.5 g/dl
Platelets	117000/ul	150000-450000/ul
Hematocrit	83.6 fl	76-96 fl
HbsAg (by ICT)	Negative	Negative
Anti-HCV (by ICT)	Negative	Negative
Serum creatinine	1.41 mg /dl	0.3-0.9 mg/dl
ALT	20 U/L	10-50 U/L
ESR	80 mm /first hour	0-20 mm /first hour

**Table 2:** Routine urine exam. RBC: red blood cells; WBC: white blood cells, R.E.: routine examination, HPF: high-powered field.

Urine R.E	Value	Reference range
Color	Yellow	Yellow
Appearance	Clear	Clear
Glucose	Nil	Negative
Protein	Positive (+)	Negative
PUS/WBC	8-10	0-5/HPF
RBC	1-2	0-5/HPF
Epithelial cells	Positive (+)	0-10/HPF
Hyaline cast	Positive (+)	0-4/HPF
Granular cast	Positive (+)	0/HPF
Amorphous urates	Nil	Nil

**Table 3:** 24 hour urinary protein.

Protein-24 hours urine	Value	Reference range
Volume	800 ml	1500-2000 ml
24 hours urinary protein	438 mg/24 hours	<150 mg/24 hours

## Discussion

In SLE, thrombotic and inflammatory phenomena can affect the eye. They can cause a range of ocular manifestations, like dry eyes, scleritis, uveitis, ischemic optic neuropathy, and occlusion of retinal vessels. Central retinal artery occlusion (CRAO) presenting in SLE is very uncommon.

There are three types of direct retinal damage in lupus, i.e., microangiopathy, vaso-occlusion, and vasculitis. The major pathology of lupus retinopathy is attributed to vasculopathy, most commonly microangiopathy [8]. The most severe form of life-threatening vision loss is vaso-occlusion, ischemia from blockage of the central retinal artery, as was the case with our patient. Vaso-occlusive retinopathy has a poor prognosis and visual losses are reported in 80% of cases [9]. Posterior segment involvement of the retina can precede systemic features of SLE and may aid in diagnosing and promptly treating patients with SLE, but it is very uncommon [10]. There is also a strong association between this type of lupus retinopathy and antiphospholipid syndrome [11]. Antibodies for antiphospholipid syndrome were negative in our patients; however, they are found in only approximately 30% of SLE patients [12].

Our patient had signs and symptoms suggestive of SLE, namely, polyarthritis, alopecia, malar rash, and Raynaud phenomena, but since she was never diagnosed and had never taken treatment for SLE, sudden vision loss prompted a visit to an ophthalmologist. Physical exam findings raised a suspicion of underlying connective tissue disorder, and the patient was referred to a rheumatologist. She was started on methylprednisone pulse therapy, 500 mg once a day for three days, antiplatelet, hydroxychloroquine, and vasodilators, but she showed little to no improvement in her visual acuity. As mentioned before, vaso-occlusive retinopathy has a poor prognosis. In one case report by Radosavljević et al., a female patient with SLE and associated retinopathy failed to improve even after aggressive steroids and immunosuppressants, and on a four-year follow-up, the disease progressed to retinal neovascularization [13]. 

Treatment of SLE involves multiple drugs like anti-malarial, steroids, immunosuppressants, biologics, and IV immunoglobulins. Still, no consensus exists on treating retinal vaso-occlusive disease in SLE. Antiplatelets and anti-lipids are usually recommended, but the role of steroids and immunosuppressants in retinal vaso-occlusive disease is unclear, and studies have suggested no such particular role in the management [14,15].

The occurrence of CRAO in SLE patients is rare nowadays because of available treatment, but since our patient had never been diagnosed with SLE and only sudden onset vision loss prompted her emergency visit that ultimately led to the diagnosis of SLE.

## Conclusions

This case highlights the critical need for early recognition and comprehensive management of systemic lupus erythematosus (SLE), particularly in patients presenting with sudden vision loss and systemic symptoms. Central retinal artery occlusion (CRAO) in SLE is a rare but severe manifestation indicative of underlying vasculopathy and often carries a poor prognosis despite aggressive treatment. The absence of prior SLE diagnosis in our patient underscores the importance of considering SLE in differential diagnoses for acute vision loss. Prompt diagnosis and appropriate multidisciplinary management are essential to mitigate severe complications and improve patient outcomes in SLE.
